# ECPR for out-of-hospital cardiac arrest: more evidence is needed

**DOI:** 10.1186/s13054-019-2722-0

**Published:** 2020-01-07

**Authors:** Graeme MacLaren, Amirali Masoumi, Daniel Brodie

**Affiliations:** 1 Cardiothoracic Intensive Care Unit, National University Health System, 5 Lower Kent Ridge Rd., Singapore, 119074 Singapore; 2 Paediatric Intensive Care Unit, Department of Paediatrics, The Royal Children’s Hospital, University of Melbourne, Flemington Rd., Parkville, VIC 3052 Australia; 3 Columbia University College of Physicians and Surgeons/New York-Presbyterian Hospital, New York, USA

**Keywords:** Extracorporeal membrane oxygenation, Extracorporeal life support, Adult, Mobile, Field, Extracorporeal cardiopulmonary resuscitation, ECMO, ECLS, ECPR

The use of extracorporeal membrane oxygenation during cardiac arrest (extracorporeal cardiopulmonary resuscitation (ECPR)) has increased in recent years [1] after evidence emerged that it was associated with better outcomes than conventional CPR for in-hospital cardiac arrest [2–4]. This success led some clinicians to attempt ECPR in highly selected patients who suffered out-of-hospital cardiac arrest (OHCA), often cannulating them on arrival in the emergency department [5]. One key determinant of the likelihood of survival in ECPR patients is the duration of CPR prior to cannulation [2, 3, 6, 7], so investigators inferred that the outcomes for OHCA patients might be improved by cannulation in the field (*prehospital* ECPR), thereby reducing the period of inadequate circulation. However, the logistic barriers to prehospital ECPR are formidable, including the difficulties inherent to undertaking complex medical procedures in a field setting, minimizing delays in cannulation without being indiscriminate about patient selection, as well as the resource consumption. Nonetheless, some hospital networks have created mobile intensive care units with prehospital ECPR capabilities [5].

The largest study to date on the use of ECPR for OHCA was recently published, shedding new light on the effectiveness of this approach. Bougouin et al. [8] reported on 13,191 OHCA cases in metropolitan Paris. Of the 12,396 patients managed with conventional CPR, 1061 (8.6%) survived to hospital discharge, compared with 44 (8.4%) of 523 ECPR patients. ECPR was attempted but failed in 58 (11%) patients. Factors associated with survival in the ECPR group included an initial shockable rhythm and transient return of spontaneous circulation (ROSC) prior to ECPR. Of note, prehospital ECPR was associated with both higher survival and more favourable neurological outcomes (OR 2.9, 95%CI 1.5–5.9, *p* = 0.002, and OR 2.9, 95%CI 1.3–6.4, *p* = 0.008, respectively) than in those patients receiving ECPR after arrival to hospital, only 7% of whom survived compared to 15% of prehospital ECPR patients.

This study represents a significant setback to enthusiasts looking to use mechanical circulatory support as a means of addressing the poor outcomes seen in patients suffering from OHCA. The fact that there were no statistically significant differences in hospital mortality between those treated with ECPR and those treated with conventional CPR mandates a reappraisal of ECPR in OHCA patients. The study had a number of strengths, including its sheer size, the practical experience of these teams in facilitating rapid deployment ECPR [5], and its multicentre observational design, providing ‘real-world’ data.

However, there were limitations to the study, most notably the selection bias. ECPR was not initiated per protocol but rather at the discretion of individual clinicians, and therefore influenced by both known and unknown confounders. This was reflected in the difference in baseline characteristics of the ECPR patients, who were younger and more likely to receive bystander CPR (81% vs 49%, *p* < 0.001) yet, importantly, were also more likely to have CPR duration exceed 30 min (99% vs 77%, *p* < 0.001). The authors attempted to control for known confounders but were unable to demonstrate that ECPR was associated with improved hospital survival either on multivariate analysis (OR 1.3, 95%CI 0.8–2.1, *p* = 0.24) or propensity matching (OR 0.8, 95%CI 0.5–1.3, *p* = 0.41). There were a number of different groups in the study, including those with non-shockable rhythms and those without ROSC. It is possible that ECPR yields different outcomes in these various subgroups, and this may benefit from more focused study. Most importantly, the long-term quality of life and neurological outcomes were not studied. There have been calls to move beyond in-hospital mortality as the primary outcome measure in ECPR and examine more robust outcomes, such as long-term survival with adequate neurological and functional recovery [9, 10].

There is an obvious discrepancy between the outcomes reported in this study and some single-centre studies (Table 1). For example, in one study from Australia of patients with cardiac arrest managed with a combination of hypothermia, ECPR and early reperfusion, over 50% of patients survived to hospital discharge with good neurological function, including 45% of those with OHCA, although not all of the latter actually received ECPR [11]. However, patient numbers were low and other larger studies have shown comparable results to those of Bougouin et al., with survival below 10% [6]. Nonetheless, it is likely that the geographical setting has an impact on outcomes. The immediacy of bystander CPR, the resources available to those providing prehospital care, the speed with which ECPR may be initiated, local traffic congestion, and the distances between the location of OHCA victims and suitably equipped hospitals all may influence results. The skill and experience of the team likely also influence the rate of serious complications as well as ultimate outcomes.

**Table 1:** Selected outcomes with ECPR

References	Patient population	No. of ECPR patients	Survival to hospital discharge (%)
ELSO Registry [1]	IHCA + OHCA*	6994	29
Chen et al. [2]	IHCA	59	29
Shin et al. [3]	IHCA	85	35 (28-day survival)
Wengenmayer et al. [6]	IHCA OHCA	74 59	19 9
Stub et al. [11]	IHCA OHCA	15 9	60 33
Bougouin et al. [8]	OHCA	525	8

*ECPR* extracorporeal cardiopulmonary resuscitation, *ELSO* Extracorporeal Life Support Organization, *IHCA* in-hospital cardiac arrest, *OHCA* out-of-hospital cardiac arrest

*Predominantly IHCA

Is it time to call for a moratorium on ECPR in OHCA patients outside of clinical trials? Survival after ECPR for in-hospital cardiac arrest patients is approximately 25–30% [1, 2], which already places a significant financial and emotional burden on families and healthcare teams. If survival after ECPR for OHCA is genuinely below 10%, this burden may become crippling. Whether or not a healthcare system wishes to deploy ECPR for OHCA also raises questions about resource management and distributive justice [12, 13]. Should public healthcare systems channel vast resources into providing scaled-up prehospital ECPR or rather into effective public health campaigns aimed at reducing cardiovascular morbidity or improving bystander CPR?

The next step is to conduct a randomized trial comparing prehospital ECPR to conventional care, although it must be acknowledged that such studies are extremely difficult to perform. Randomized trials are already underway but are generally initiating ECPR on arrival to hospital (e.g. Clinicaltrials.gov identifiers NCT03101787, NCT03065647, NCT01605409). It is also important to study other medical interventions alongside ECPR such as early coronary revascularization [11, 14]. The authors [8] suggested that ECPR for OHCA should be restricted to patients with shockable rhythms who achieve transient ROSC. This is important, given that shockable rhythms are a surrogate for the potential for revascularization. We agree and recommend that ECPR not be used routinely in OHCA patients outside of clinical trials. To do otherwise may invite an increase in unsalvageable patient admissions associated with higher costs of care, rising clinician burnout and an unjustified burden placed on families and patients.

## Data Availability

N/A
